# Choroidal Metastasis Presenting As Anterior and Posterior Scleritis: A Rare Manifestation of a Previously Unknown Metastatic Cutaneous Melanoma

**DOI:** 10.7759/cureus.89529

**Published:** 2025-08-07

**Authors:** Daniel R Chow, Mélanie Hébert, Evangelina Esposito, Mohammed Al Kaabi, Marie-Josée Aubin

**Affiliations:** 1 Ophthalmology, McGill University, Montreal, CAN; 2 Department of Ophthalmology, Hôpital du Saint-Sacrement, Quebec, CAN; 3 Department of Ophthalmology, Hôpital Maisonneuve-Rosemont, Montreal, CAN; 4 Department of Social and Preventive Medicine, Université de Montréal, Montreal, CAN

**Keywords:** choroidal metastasis, cutaneous melanoma, masquerade, nras mutation, scleritis

## Abstract

Choroidal metastasis from occult cutaneous melanoma is rare and can masquerade as ocular inflammation. A 70‑year‑old man with sectoral anterior scleritis was found on multimodal imaging to have a solitary choroidal mass with mild periscleral fluid, prompting systemic evaluation that uncovered colonic polyps that, on histopathology, contained metastatic melanoma, a scalp primary, and widespread visceral, nodal, and intracranial metastases. Tumour cells stained HMB‑45, Melan‑A, and SOX10 positive, AE1/AE3 negative, and carried an NRAS‑Q61 mutation with wild‑type BRAF, confirming cutaneous origin. Combined immune‑checkpoint blockade and ocular‑plus‑cerebral radiotherapy preserved Snellen visual acuity of 20/50-2 (≈0.44 logMAR) in the affected eye, although systemic disease progressed four months later. This case highlights the importance of meticulous fundus examination, multimodal imaging, and molecular profiling for distinguishing metastatic choroidal melanoma from primary uveal melanoma when ocular inflammation obscures malignancy, thereby enabling timely, targeted therapy.

## Introduction

Intraocular metastases predominantly arise in the posterior choroid and may be asymptomatic or cause visual disturbances such as decreased acuity, scotomata, flashes, and floaters [1]. Less frequently, metastases can involve the anterior uveal tract and may present clinically as pain or scleritis [1-3]. These rare presentations may cause diagnostic delay and adversely affect prognosis. Because metastatic spread often precedes ocular symptoms, any delay in recognising these atypical signs can significantly shorten overall survival. As such, one should consider metastatic disease in patients with a history of cancer who present with vision changes or ocular inflammation [2,3].

The most common primary tumor sites are breast cancer in females and lung cancer in males [1,3,4]. Intraocular metastasis from a primary cutaneous melanoma is very rare (1%)[5] and usually presents after the diagnosis of disseminated metastases [6,7]. Intraocular metastatic choroidal melanoma with unknown primary site must be distinguished from uveal melanoma, the most common intraocular tumor in adults.

We describe an unusual case of choroidal metastasis from an unknown primary cutaneous melanoma, presenting with anterior and posterior scleritis. This case highlights the potential for ocular tumors to masquerade as scleritis. It also highlights the challenge in determining if the uvea is the primary or secondary site in patients with a yet undiagnosed cancer.

## Case presentation

A 70-year-old male with a family history of colon cancer, but no personal history of neoplasms, presented with a one-month history of unilateral ocular redness and boring scleral pain with no photophobia. On initial examination, visual acuity was 20/50+2 in the right eye (OD) (≈0.36 logMAR) and 20/20-1 in the left eye (OS) (≈0.02 logMAR), and intraocular pressure was 18 mmHg OD and 12 mmHg OS, respectively.

**Figure 1:**
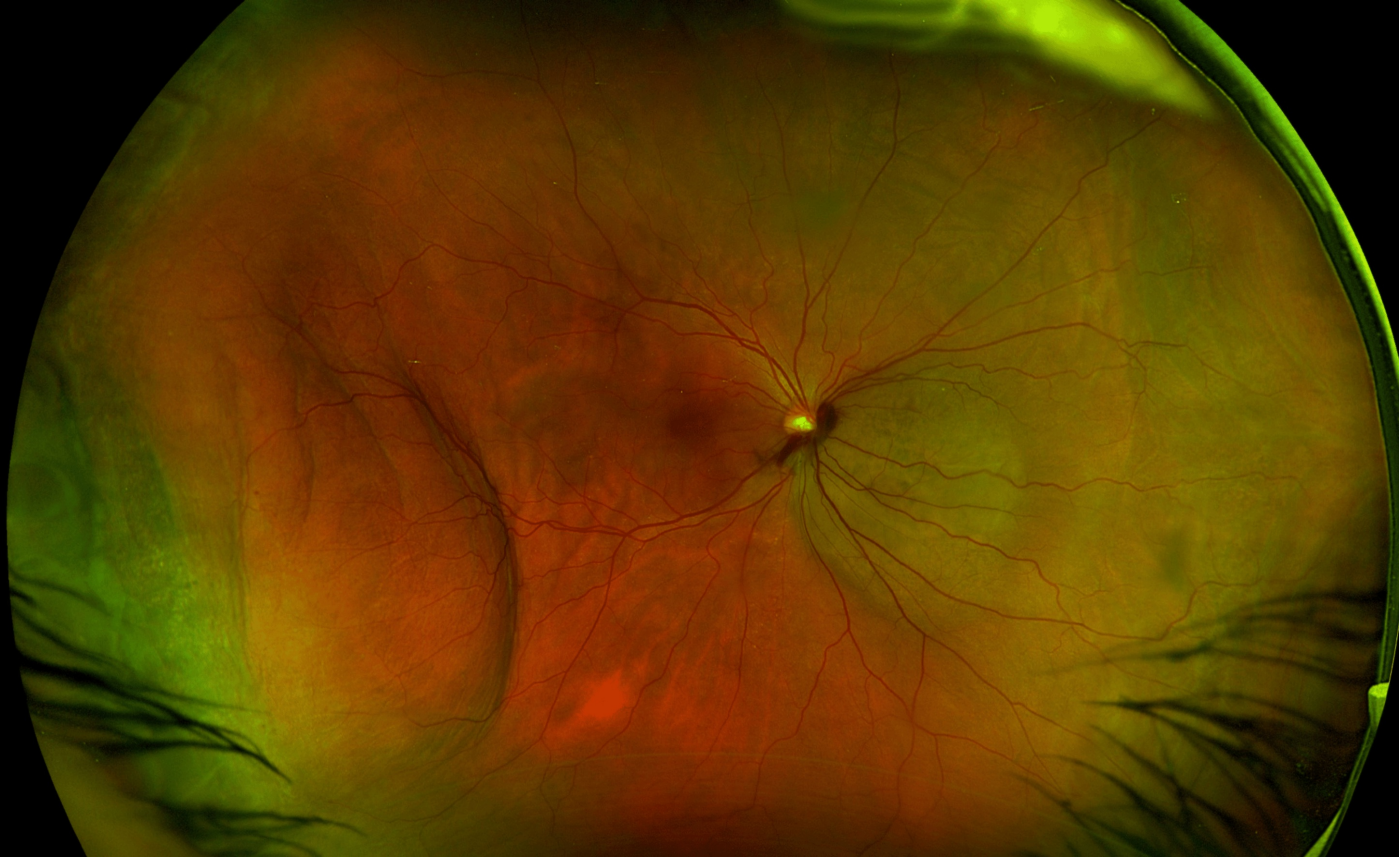
Pseudocolour optos wide-field fundus image (right eye) showing a posterior pole, non-pigmented choroidal lesion nasal to the optic disc, corresponding to the choroidal metastasis, and concomitant peripheral choroidal detachment temporally

The OD anterior segment showed dilated and tortuous episcleral and deep scleral vessels in the nasal and superonasal quadrants, painful upon palpation, consistent with a diagnosis of sectorial anterior scleritis. The anterior chamber was clear with no cells or flare, confirming the absence of anterior uveitis. The OS anterior segment was unremarkable. Fundus examination of the OD revealed a non-pigmented choroidal mass nasal to the optic disc, of approximately 5 disc diameters (DD) by 2 DD in basal dimensions and 4 DD (6 mm) in height, with peripheral serous choroidal detachments (Figure 1). Fluorescein fundus angiography (FFA) showed some hyperfluorescence of the lesion with late staining. The fundoscopy and FFA of OS were unremarkable aside from a small previously treated retinal tear. Ultrasonography confirmed a 13.6 mm basal diameter x 5.9 mm height choroidal mass nasal to the optic nerve OD. This lesion was nodular with heterogeneous internal structures on A and B modes. B-scan did not demonstrate the classic 'T sign'; however, it showed mild periscleral fluid and focal choroidal detachment without diffuse choroidal thickening, findings consistent with concomitant posterior scleritis [8]. No exudative (serous) retinal detachment was present; the observed serous detachments were confined to the choroid. With the presence of a solitary choroidal nodular mass, the diagnoses considered were primary choroidal amelanotic melanoma or choroidal metastasis from a yet unknown primary.

A uveitic work-up was undertaken to exclude infection. The patient was treated with topical prednisolone acetate 1% ophthalmic suspension, one drop every two hours while awake for seven days, then tapered to four times daily in week two, twice daily in week three, once daily in week four, and stopped, together with atropine sulfate 1% one drop twice daily for two weeks, and oral prednisone 60 mg daily for five days followed by weekly tapering to 50 mg, 40 mg, 30 mg, 20 mg, and 10 mg to control the scleritis.

Because of the patient's familial history of colon cancer, a colonoscopy was performed. A pathological diagnosis of metastatic melanoma was reached from polyp biopsies. A dermatology evaluation revealed a suspicious scalp lesion that was biopsied. Immunohistochemistry showed strong positivity for HMB-45, Melan-A, and SOX10 and negativity for pancytokeratin AE1/AE3. Targeted sequencing identified an NRAS-Q61 mutation with wild-type BRAF. A positron emission tomography (PET) scan revealed numerous hypermetabolic, hypodense hepatic lesions and multicentric metastatic involvement in infra-diaphragmatic, hepatic, splenic, muscular, and osseous lymph nodes. Later, cerebral and orbital magnetic resonance imaging (MRI) confirmed the OD choroidal mass and revealed multiple intracranial lesions with potential hemorrhagic components. Based on these findings, a diagnosis of anterior and posterior scleritis from a choroidal melanoma metastasis in the context of metastatic disease from a primary cutaneous (scalp) melanoma was established. The patient was referred to oncology for specialized care.

Following two cycles of immunotherapy, metastatic progression persisted, along with headache, scotoma, and a three-day episode of blurred vision OD. Examination of the OD revealed vitreous hemorrhage, and MRI showed slight growth of the choroidal mass. On final follow-up, visual acuity was 20/50-2 OD (≈0.44 logMAR) and 20/20 OS (0.00 logMAR), with a slight progression of the choroidal mass OD but no signs of scleritis or intraocular inflammation. In addition to immunotherapy, the patient was offered local ocular radiotherapy, aiming to reduce lesion size, preserve vision, and minimize discomfort.

## Discussion

Choroidal metastasis occurs in a small but significant proportion (2-7%) of cancer patients [1]. Most are associated with invasive ductal carcinoma in females and lung adenocarcinoma in males. A cutaneous primary is very rare [5]. Furthermore, less than 10% of all melanoma cases present with unknown primary tumor location, as in the case described herein, where the primary source, initially unknown, was found to be cutaneous melanoma [1,3,4,9]. Distinguishing a primary uveal melanoma from a secondary choroidal deposit is clinically pivotal yet challenging. Primary tumors are usually solitary, dome-shaped lesions with orange lipofuscin and medium-to-low ultrasound reflectivity, whereas metastatic melanoma tends to be flatter, multifocal, or bilateral, and shows higher internal acoustic reflectivity [10].

Clinical examination, including dilated fundoscopy, is the cornerstone for diagnosing ocular metastasis. In complex cases, as those with unknown primary, multimodal imaging (e.g., FFA, optical coherence tomography, ocular ultrasonography) and full-body imaging can help establish a definitive diagnosis. Biopsy, including fine needle aspiration biopsy (FNAB), is often essential, although it carries a small but real risk of intraocular or retinal complications [1-3]. In patients with metastasis of unknown primary, histopathological evaluation requires careful interpretation of immunohistochemistry biomarkers to identify the organ of origin and molecular make-up. The immunophenotype in our case, positivity for HMB-45, Melan-A, and SOX10 with absence of pancytokeratin, confirmed melanocytic lineage while helping to exclude carcinoma. NRAS is only rarely mutated in uveal melanoma, so an NRAS-positive/BRAF-wild-type genotype points to a cutaneous origin [11]. In cutaneous melanoma, >80% of NRAS mutations occur at Q61 (exon 2) or, less often, at codon 12 (exon 1) and co-exist with BRAF V600E in <1% of treatment-naïve tumors [11-14].

Differentiating primary uveal melanoma with metastasis from primary cutaneous melanoma with choroidal metastasis is important as their treatment and prognosis differ [13-15]. Although both carry a high morbidity and mortality rate, survival rates are generally better for patients with cutaneous melanoma than for those with uveal melanoma [13-15].

The prognosis of choroidal metastasis is generally poor, with a mean survival of 4-12 months [1]. Treatment options focus on palliative care based on various factors such as the physical condition of the patient, the location and number of primary tumors, and intraocular metastases, as well as distant metastases [1]. In this case, the patient was offered palliative ocular radiotherapy as a means of limiting local progression of the tumor and preserving quality of life.

## Conclusions

Ocular findings, however subtle or atypical, should prompt consideration of tumoral etiology. This case highlights the potential for ocular metastasis to be the first sign of malignancy, prompting systemic evaluation and identification of the primary site. The ability of neoplasms to mimic inflammatory conditions such as scleritis underscores the need for careful evaluation and thorough investigation of anterior segment inflammation with proper fundus examination. Differentiating primary choroidal melanoma from melanoma metastasis can be challenging; increased awareness of atypical presentations is essential for prompt diagnosis and treatment.
